# Clinical features and management of manic episodes in adolescents. A case review

**DOI:** 10.1192/j.eurpsy.2022.1128

**Published:** 2022-09-01

**Authors:** V.E. Martin Gil, M.C. Chinchilla Moreno

**Affiliations:** 1 Hospital Universitario Virgen del Rocío, Ugc Salud Mental, Sevilla, Spain; 2 Hospital Universitario Gregorio Marañón, Psiquiatría, Madrid, Spain

**Keywords:** bipolar disorder, manic episode, bipolar disorder treatment, Adolescents

## Abstract

**Introduction:**

Assessment and management of bipolar disorder, and particularly manic episodes in adolescents means a challenge. The presence of comorbid disorders, and divergent interpretations of manic symptoms in the context of the adolescent natural inmaturity, can make diagnosis and treatment hard goals to achieve. The existence of juvenile specific criteria for bipolar disorder is a debate topic. This concept emerged from an attempt to solve diagnosis issues and involves a wide range of definitions for mania.

**Objectives:**

Literature review concerning bipolar disorder in young population: Main comorbidities, psychosocial problems, prognosis. Clinical presentation: Shared and specific features compared to adult population. Available treatment options. Issues related to safety and tolerability.

**Methods:**

We present a case of a 16 year old woman diagnosed with bipolar II disorder, hospitalised in an inpatient adolescent unit in 2021. Review of the literature available (clinical guidelines, PubMed).

**Results:**

Patient initially oriented as a Bipolar II disorder, after depressive episodes followed by hypomanic symptoms in the past years. The following clinical course was conditioned by personality traits. Emotional disregulation and a complex family environment made affective symptoms difficult to evaluate, leading to a diagnostic hypothesis of personality-related disorder. After a period of outpatient treatment in a day hospital, she debuted with a clinical picture of manic symptoms, mixed features and rapid mood cycling.

**Conclusions:**

After an initial trial, stabilization was achieved with aripiprazole and asenapine. Combination therapy might be necessary in longer-term treatment, according to existing evidence. Diagnosis and treatment concerns are interfered by the limited number of trials.

**Disclosure:**

No significant relationships.

